# Immunoglobulin domain interface exchange as a platform technology for the generation of Fc heterodimers and bispecific antibodies

**DOI:** 10.1074/jbc.M117.782433

**Published:** 2017-04-27

**Authors:** Darko Skegro, Cian Stutz, Romain Ollier, Emelie Svensson, Paul Wassmann, Florence Bourquin, Thierry Monney, Sunitha Gn, Stanislas Blein

**Affiliations:** From the ‡ Department of Antibody Engineering, Biologics Research, Glenmark Pharmaceuticals S.A., Chemin de la Combeta 5, 2300 La Chaux-de-Fonds, Switzerland and; the § Department of Drug Metabolism and Pharmacokinetics, Glenmark Pharmaceuticals Limited, Glenmark Research Centre, Plot No. A-607, T.T.C. Industrial Area, MIDC, Mahape, Navi Mumbai 400 709, India

**Keywords:** antibody, antibody engineering, monoclonal antibody, protein engineering, T-cell receptor (TCR), CH3, bispecific, exchange, heterodimer, interface

## Abstract

Bispecific antibodies (bsAbs) are of significant importance to the development of novel antibody-based therapies, and heavy chain (Hc) heterodimers represent a major class of bispecific drug candidates. Current technologies for the generation of Hc heterodimers are suboptimal and often suffer from contamination by homodimers posing purification challenges. Here, we introduce a new technology based on biomimicry wherein the protein-protein interfaces of two different immunoglobulin (Ig) constant domain pairs are exchanged in part or fully to design new heterodimeric domains. The method can be applied across Igs to design Fc heterodimers and bsAbs. We investigated interfaces from human IgA CH3, IgD CH3, IgG1 CH3, IgM CH4, T-cell receptor (TCR) α/β, and TCR γ/δ constant domain pairs, and we found that they successfully drive human IgG1 CH3 or IgM CH4 heterodimerization to levels similar to or above those of reference methods. A comprehensive interface exchange between the TCR α/β constant domain pair and the IgG1 CH3 homodimer was evidenced by X-ray crystallography and used to engineer examples of bsAbs for cancer therapy. Parental antibody pairs were rapidly reformatted into scalable bsAbs that were free of homodimer traces by combining interface exchange, asymmetric Protein A binding, and the scFv × Fab format. In summary, we successfully built several new CH3- or CH4-based heterodimers that may prove useful for designing new bsAb-based therapeutics, and we anticipate that our approach could be broadly implemented across the Ig constant domain family. To our knowledge, CH4-based heterodimers have not been previously reported.

## Introduction

The need to improve the clinical efficacy of antibodies has led to an increased interest in bsAbs^4^ (1–4). bsAbs combine specificities of two antibodies in a single antibody construct that is able to bind two different epitopes on the same or on different antigens. Among the multitude of bsAb formats, the Hc heterodimer format is found in a majority of drug candidates as this Fc-containing architecture intrinsically benefits from a long serum half-life and the ability to mediate effector functions (5). It allows the design of asymmetric bsAbs wherein two different antigen-binding arms are, respectively, part of two different Hc chains that heterodimerize instead of forming homodimers, usually through a pair of engineered CH3 domains.

Hc heterodimerization (HD) was first reported by Atwell *et al.* (6) using a technique known as “knobs-into-holes” (KiH). The technology is based on an engineered pair of CH3 domains that heterodimerizes (CH3 heterodimer) and involves introducing mutations that create a protuberance in the interface of the first CH3 domain and a corresponding cavity in the interface of the second CH3 domain, such that the protuberance can be positioned in the cavity to promote heterodimer assembly and hinder homodimer formation. Developed in the mid-1990s, large scale expression and production of KiH-based bsAbs in mammalian cells have been reported to be challenging due to variable heterodimer purity (7). Furthermore, KiH was initially hampered due to the random light chain (Lc) association inherent to the method. Originally, the use of a common Lc derived from phage display screens was proposed but never broadly reported (8, 9). A more recent solution involves a domain crossover between heavy and light constant domains within one of the Fab arms of the bsAb, thereby enforcing correct Lc pairing (10).

Over the last 6 years, several solutions to create CH3 heterodimers have been proposed, most of which have emerged from rational design (11–13). An example of a more systematic approach to design Hc heterodimers is found in the SEED technology (14). SEED is based on an exchange of β-strands and loops between IgA and IgG1 CH3 homodimers to create a new CH3 heterodimer. Although the technology allows efficient generation of Hc heterodimers, one drawback is that the resulting CH3 heterodimer includes >50 amino acid changes and six amino acid insertions thereby increasing the risk of immunogenicity.

Teachings from the current Hc HD technologies helped us identify three key bottlenecks as follows: (i) CH3 heterodimers that do not heterodimerize efficiently upon co-expression in mammalian cells and yield low bispecific content, a bottleneck that has motivated the development of Hc heterodimers in *Escherichia coli* (7); (ii) contamination by homodimers occurring upon scale-up (12); and (iii) Lc mispairing, an issue that is usually addressed with a common Lc isolated from hybridoma screens (12, 15) or a case-by-case re-engineering of the two parental Fabs (16). To address the present shortcomings, we first set out to design an efficient Hc HD technology; we then implemented a platform purification solution to remove any homodimer traces that may occur at scale-up, and finally we used an scFv × Fab format for the two binding arms to successfully address Lc mispairing. Importantly, the resulting bsAb is the secretion product of one mammalian cell line thus minimizing costs and effort.

**Figure 1.**
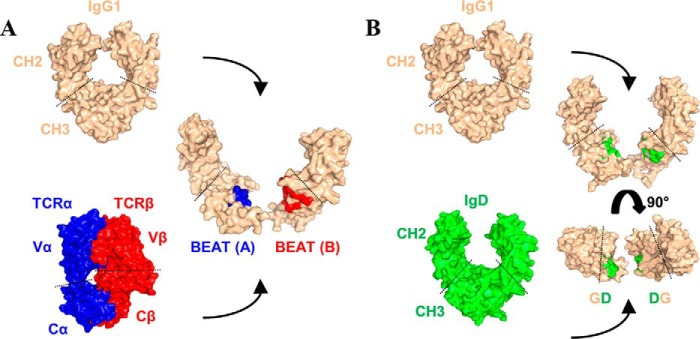
**New heterodimeric interfaces can be built in two different ways.** *A,* using heterodimers as donor interfaces, BEAT: a heterodimeric interface, in this case that of TCR Ca-Cb, is grafted onto a homodimeric interface such as that of IgG1 CH3. *B,* mixing homodimer interfaces, half of a homodimeric interface, in this case that of IgD CH3, is grafted onto another homodimeric interface such as that of IgG1 CH3.

We started our engineering work by looking at 3D structures of Ig domain pairs found in various proteins of the immune system. Ig constant domains found in antibodies share the same tertiary fold but also assemble into very similar quaternary structures. TCRs are striking examples of these similarities extending outside the antibody family with their 3D structures being almost equivalent to the Fab portion of an antibody. As the Ig constant domain fold is strongly conserved, we saw in quaternary structures of antibodies and TCRs a source of protein-protein interfaces that could be used entirely or partially to engineer new heterodimers. By exchanging residues from the IgG1 CH3 homodimer interface with residues from the TCR α/β constant domain (TCR Ca-Cb) interface (Fig. 1*A*), we created a new CH3 heterodimer that heterodimerizes more efficiently than the previously described KiH and SEED technologies. Following this initial work, we succeeded in engineering additional heterodimers based on the TCR γ/δ constant domain (TCR Cg-Cd) interface or by exchanging half-interfaces between two Ig homodimers (Fig. 1*B*). In total, we report the successful design of five new CH3 heterodimers and two new CH4 heterodimers along with methods to systematically exchange interfaces between Ig constant domain pairs using the IMGT numbering system (17, 18). To further validate our concept of interface exchange, we solved the crystal structure of our initial CH3 heterodimer and demonstrated the successful importation of the TCR Ca-Cb interface within the IgG1 CH3 domain pair.

As a proof-of-concept for the design of future therapeutic bsAbs, we used our TCR-based HD technology or BEAT platform (Bispecific Engagement by Antibodies based on the T-cell receptor) to engineer and produce examples of bsAbs or BEATs that target growth factor receptors broadly involved in oncology.

## Results

### Hc HD technology based on an interface exchange between TCR and antibody Ig constant domains

The IMGT database was used to identify the protein-protein interface residues located in the β-strands of various human Ig constant domain pairs found in antibodies and TCRs (http://www.imgt.org/IMGTrepertoire/Proteins/; section: C-DOMAIN).^5^ The IMGT numbering system for C-DOMAIN provides a link between the 3D structures of Ig constant domains and their amino acid sequences, thus allowing the identification of equivalent 3D positions between domains. For all homo- and heterodimers from C-DOMAIN, we identified the following positions as interface residues: 3, 5, 7, 20, 22, 26, 27, 79, 81, 84, 84.2, 85.1, 86, 88, and 90.

**Figure 2.**
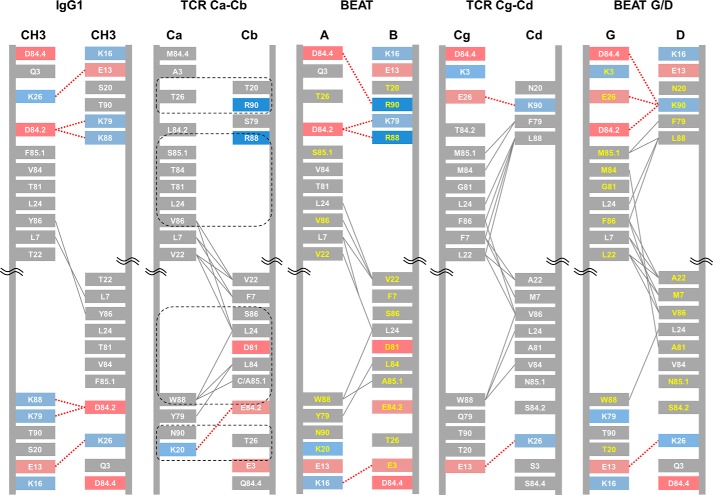
**Schematic diagrams depicting the interfaces of IgG1 CH3, TCR constant domain pairs, and BEAT CH3 interfaces.** The interdomain interactions were calculated from solved structures or models. The IMGT numbering is used. Charged residues are colored in *red* (negative) or *blue* (positive). Hydrophobic interactions are in *gray lines*, and electrostatic interactions in *dashed red lines*. Grafted residue numbers are in *yellow*. The four key sets of residues selected for grafting the TCR Ca-Cb interface onto the IgG1 CH3 homodimer are *circled* in *dashed black lines*.

We first attempted to exchange the IgG1 CH3 homodimer interface with the TCR Ca-Cb interface (Fig. 1*A*). From the analysis of the TCR Ca-Cb interface (Fig. 2), we identified four groups of residues that could possibly disrupt the CH3 interface symmetry and recreate some TCR contacts upon grafting (two pairs of reciprocal sets). The first group is composed of Trp 88 in TCR Ca with positions Ala 85.1 and Ser 86 in TCR Cb. These positions were selected to recreate the Trp 88 (TCR Ca)/Ala 85.1 (TCR Cb) hydrophobic interaction and disrupt the Tyr 86 symmetric hydrophobic contact found in IgG1 CH3. Note that position 85.1 in the natural TCR Cb sequence corresponds to a cysteine, but we used alanine to prevent disulfide bond mispairing. The second set of residues includes position Arg 88 in TCR Cb with positions Ser 85.1 and Val 86 in TCR Ca. Arg 88 was considered to be a silent change that will maintain the original network of electrostatic contacts at Lys 88 in IgG1 CH3. We postulated that the introduction of Trp 88 from TCR Ca in the other CH3 monomer would have disrupted the symmetric network of electrostatic contacts found on the opposite side of the interface; thus, maintaining this network of contacts creates asymmetry. The other substitutions were selected to disrupt the Tyr 86 symmetric hydrophobic contact mentioned above (Val 86) and to recreate the Val 86 (TCR Ca)/Val 22 (TCR Cb) hydrophobic contact with the help of supporting position Val 22 in TCR Cb. Position Ser 85.1 was selected for grafting to spatially accommodate the import of the other two positions. The third group of residues is composed of position Lys 20 in TCR Ca with position Thr 26 in TCR Cb. Lys 20 in TCR Ca was imported to putatively create a new electrostatic contact with supporting position Glu 84.2, although this interaction was only seen in one of the TCR Ca-Cb structures we used for analysis (PDB code 1KGC), and Thr 26 in TCR Cb was selected to disrupt the Lys 26/Glu 13 symmetric electrostatic contact found in IgG1 CH3. Finally, the fourth set consists of position Thr 20 in TCR Cb with position Thr 26 in TCR Ca. Thr 26 in TCR Ca was selected to disrupt the Lys 26/Glu 13 symmetric electrostatic contact discussed above but found on the opposite side of the IgG1 CH3 interface; Thr 20 in TCR Cb was a conservative change as position 20 is a serine in IgG1 CH3. These four sets of residues are supported by other TCR Ca-Cb interface positions; in both domains positions 3, 5, 7, 22, 79, 81, 84, 84.2, and 90 brought asymmetric contacts and/or helped to spatially accommodate other substitutions that were selected for exchange.

We then derived a new pair of IgG1 CH3 domains having TCR Ca-Cb-based substitutions as follows (BEAT; Fig. 2 and supplemental Fig. S1): a first CH3 domain based on TCR Ca having substitutions S20K, T22V, K26T, K79Y, F85.1S, Y86V, K88W, and T90N (BEAT CH3 (A)), and a second CH3 domain based on TCR Cb having substitutions Q3E, Y5A, L7F, S20T, T22V, K26T, T81D, V84L, D84.2E, F85.1A, Y86S, K88R, and T90R (BEAT CH3 (B)).

The resulting CH3 heterodimer was then used to create an Fc-like protein made of two Fc-like chains, each chain including one of the two engineered CH3 domains (Fc-like chain as follows: IgG1 hinge-IgG1 CH2-engineered IgG1 CH3 domain). Homo- and heterodimeric Fc-like proteins are difficult to identify as these have a similar molecular weight; thus a variable Lc κ domain antibody (VL-dAb) was N-terminally fused to one of the Fc-like chains to generate a significant difference in molecular weight. The two Fc-like chains were transfected at a 1:1 ratio into HEK293-EBNA cells. Post-Protein A purification, heterodimer formation was assessed by non-reduced SDS-PAGE and quantified by scanning densitometry of the gel bands.

**Figure 3.**
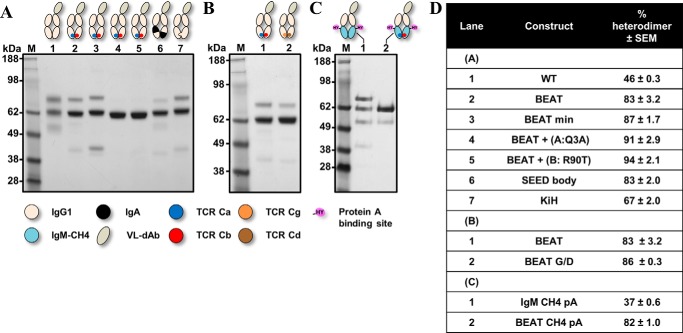
**SDS-PAGE analysis of the new CH3 and CH4 heterodimers based on the TCR constant domain interfaces.** Engineered Fc-like proteins were transiently expressed, purified by Protein A chromatography and analyzed by SDS-PAGE. *A,* BEAT Fc and variants thereof, an Fc-like protein with a CH3 heterodimer based on the TCR Ca-Cb interface; a control and Fc-like proteins based on previously described technologies are shown. *B,* BEAT Fc *versus* BEAT G/D Fc, an Fc-like protein with a CH3 heterodimer based on the TCR Cg-Cd interface. *C,* BEAT CH4 Fc, an Fc-like protein with a CH4 heterodimer based on the TCR Ca-Cb interface wherein Protein A binding was engineered by mutagenesis. The Fc-like protein IgM CH4 pA corresponds to a control Fc-like protein based on the IgM CH4 homodimer wherein Protein A binding was also engineered. *D,* summary of heterodimer content.

Fig. 3*A* shows the results of this initial TCR Ca-Cb interface graft *versus* other known Hc HD technologies. From the control IgG1 Fc (Fig. 3*A*, *lane 1*), we observed three bands that correspond to the three expected species upon random pairing of the chains: assembled heterodimer (VL-Fc/Fc, 62.7 kDa, 46%) and the two homodimers (VL-Fc/VL-Fc and Fc/Fc, 74.4 and 51 kDa, respectively). Our initial TCR Ca-Cb interface graft favored HD (Fig. 3*A*, *lane 2,* 83%) and was later redesigned with significantly fewer substitutions (BEAT min; supplemental Figs. S1 and S2): BEAT min CH3 (A) having substitutions S20K, T22V, K26T, K79Y, K88W, and T90N and BEAT min CH3 (B) having substitutions F85.1A and Y86S and exhibiting a small increase in HD with respect to our original graft (Fig. 3*A*, *lane 3*, 87%). Addition of Q3A in BEAT CH3 (A) (Fig. 3*A*, *lane 4*) or removal of T90R in BEAT CH3 (B) (Fig. 3*A*, *lane 5*) allowed HD to reach over 90% and close to 95%, respectively; this means that homodimers were not visually detectable. The HD levels observed for the SEED (Fig. 3*A*, *lane 6*) (14) and KiH (Fig. 3*A*, *lane 7*) (6, 9) technologies were significantly lower, 83 and 67%, respectively.

Another example of a natural heterodimeric interface is found in the TCR Cg-Cd domain pair. Using the same design approach, we grafted the TCR Cg-Cd interface onto the IgG1 CH3 homodimer (BEAT G/D), including the four key sets of positions described above along with the same supporting positions (Fig. 2 and supplemental Fig. S1). The TCR Cg-Cd differs from the TCR Ca-Cb as position 88 in both domains brings interdomain hydrophobic interactions; in addition, two asymmetric electrostatic contacts are present. Despite the large number of expected asymmetric contacts, grafting the TCR Cg-Cd interface only increased HD by 3% with respect to our BEAT interface (BEAT G/D; Fig. 3*B*, *lane 2*, 86%).

Next, we attempted to graft the TCR Ca-Cb interface onto another class of Ig dimer and selected the IgM CH4 homodimer. Using the same method and sets of positions, we built a CH4 heterodimer based on the TCR Ca-Cb interface (supplemental Figs. S1 and S2). For better comparability between CH3 and CH4 engineering, CH4 heterodimers were also tested in the context of an Fc-like protein wherein engineered CH3 domains were replaced by engineered CH4 domains. Because CH4 domains lack Protein A binding, substitutions R115H and V116Y (IMGT numbering) were introduced to confer binding (pA). The CH4 heterodimer (BEAT CH4 pA) exhibited a HD rate similar to that of the BEAT CH3 heterodimer (Fig. 3*C*, *lane 2*, 82%).

### BEAT Fc characterization and crystal structure

The heterodimeric Fc derived from the original BEAT interface had good transient expression and good thermal stability; the thermal stability of the BEAT Fc region was similar to that of the human IgG4 Fc region (19) as judged by its calorimetric profile showing a single transition with a *T_m_* of 70 °C (supplemental Fig. S3*A*), a melting point that is consistent with other previously reported CH3 heterodimers such as KiH (6).

**Figure 4.**
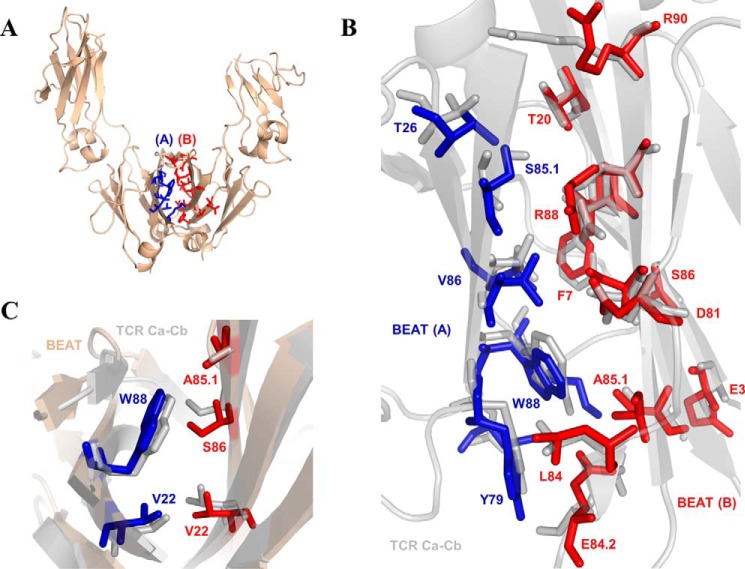
**Crystal structure of the BEAT Fc.** *A,* ribbon diagram. *B,* structural alignment of grafted residues from the BEAT interface with corresponding residues in TCR Ca-Cb (IMGT numbering). The structure of the BEAT CH3 heterodimer was superimposed on that of the TCR Ca-Cb heterodimer. For BEAT, only grafted residues are displayed at top of the TCR Ca-Cb structure (PDB code 1KGC). *C,* close-up showing the conservation of the side-chain orientation for positions 22 and 88 in monomer (A) and 22, 85.1, and 86 in monomer (B) between the BEAT and TCR interfaces. BEAT CH3 (*A*) residues are colored *blue* and BEAT CH3 (*B*) residues are colored *red*.

To further characterize this fragment, the BEAT Fc was crystallized and its 3D structure solved at high resolution (Table 1). The same so-called “horseshoe” structure as that seen in any natural IgG antibody was observed (Fig. 4*A*), with little deviation from other experimentally solved structures (root mean square deviation of 0.66 Å *versus* PDB code 2WAH when superimposing the CH3 domains on their Cα traces). Analysis of the engineered CH3 domains revealed the successful importation of key heterodimeric contacts found in the TCR Ca-Cb crystal structure (Fig. 4, *B* and *C*) with 85% of the imported TCR residues maintaining their original side chain orientation in the BEAT Fc structure. The four key sets of substitutions used in our engineering strategy were found to have the expected functions we described above (Fig. 2) and can be summarized as follows: the first set of grafted residues recreated a pair of TCR Ca-Cb hydrophobic contacts on one side of the interface (Trp 88 in (A) with Ala 85.1 and Leu 24 in (B)), whereas the second set maintained the existing network of electrostatic interactions at the opposite side thereby creating asymmetry. The third and fourth sets removed the Lys 26/Glu 13 pair of symmetric electrostatic contacts. Supporting positions 3, 7, 22, 79, 84, and 90 further contributed to break symmetry and add asymmetric contacts. Q3E in (B) created a new ionic interaction with Lys 16 in (A). L7F in (B) removed the symmetric hydrophobic interaction found at this position in IgG1 CH3. V84L in (B) created a new asymmetric hydrophobic contact with K79Y in (A), a contact directly imported from the TCR Ca-Cb interface. T22V in (B) with Leu 24 in (A) and T22V in (A) with Leu 24 in (B) created a pair of symmetric hydrophobic contacts that did not exist in the parental interfaces. Finally, T90R in (B) created a new ionic interaction with Asp 84.4 in (A), another contact that did not exist in the parental interfaces.

**Table 1:** **Data collection and refinement statistics of the solved BEAT Fc crystal structure.**

	BEAT Fc
**Data collection**	
Wavelength (Å)	0.9795
Space group	P2_1_2_1_2_1_
Cell dimensions	
*a*, *b*, *c* (Å)	49.8, 73.7, 141.5
α, β, γ (°)	90.0, 90.0, 90.0
Resolution (Å)*^a^*	30.00–1.97 (2.08–1.97)
*R*_merge_ (%)	0.119 (0.765)
*R*_meas_ (%)	0.138 (0.897)
*R*_pim_ (%)	0.069 0.458)
*I*/σ*I*	5.2 (1.3)
Completeness (%)	99.2 (98.6)
Redundancy	3.6 (3.5)

**Refinement**	
Resolution (Å)	30.00–1.97 (2.02–1.97)
No. of reflections	35360/2533
*R*_work_/*R*_free_	0.210/0.279
No. atoms	
Protein	3107
Sugar	220
Water	493
*B* factors	
Protein	54.4
Sugar	96.4
Water	59.0
Root mean square deviations	
Bond lengths (Å)	0.010
Bond angles (°)	1.694

*^a^* Values in parentheses are for highest-resolution shell.

Addition of Q3A in (A) or back mutating position 90 (R90T) in (B) enhanced HD. In light of the solved structure, we made the following observations. The substitution of Gln 3 by an alanine increases HD by putatively lowering steric hindrance due to the larger side chain of the glutamine residue. In a modeled homodimer of two BEAT CH3 (B) domains (data not shown), we observed Arg 90 in one domain making electrostatic interactions with Glu 84.2 and Asp 84.4 in the other domain (also true for the reciprocal set of residues). By back mutating Arg 90 for threonine, these interactions are removed thereby destabilizing the BEAT CH3 (B) homodimer; a similar result was obtained by substituting Asp 84.4 for a glutamine (D84.4Q).

Although the original TCR Ca-Cb graft and variants thereof introduce a significant number of substitutions, it is important to note that most substitutions are buried at the heterodimer interface with only a small number of these residues protruding and therefore solvent-exposed, some of which are found at the bottom of surface cavities (supplemental Fig. S3*B*), suggesting a low immunogenicity risk.

### Design of new heterodimers by mixing homodimeric interfaces

The initial success of our TCR interface exchange method led to further investigations into designing new heterodimers from two existing homodimers (Fig. 1*B*). Exchanging half of an acceptor homodimer interface with the equivalent half-interface of a donor homodimer was thought to be sufficient for breaking symmetry and generating a new heterodimer. To this end, the IgG1 CH3 interface (Fig. 2) was grafted with half of the interface of either human IgA CH3, IgD CH3, or IgM CH4 (supplemental Fig. S2 and S4), whereas the IgM CH4 interface was grafted with half of the interface of IgG1 CH3.

**Figure 5.**
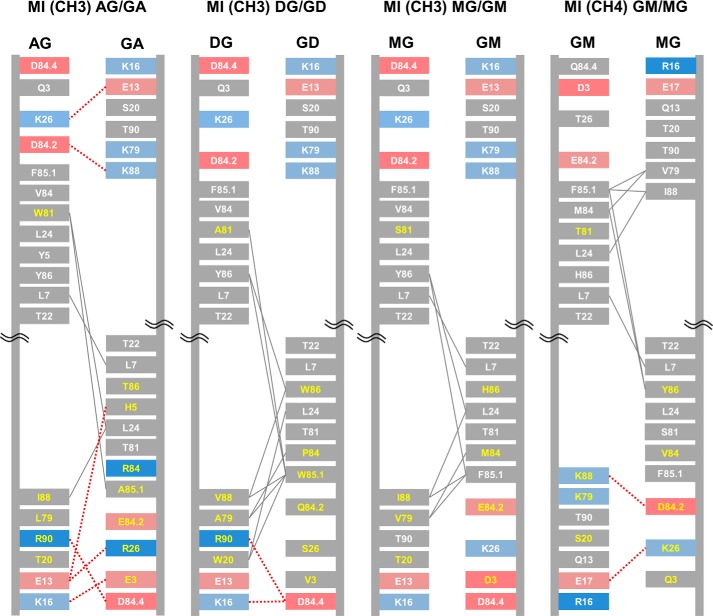
**Schematic diagrams depicting the interfaces of the new CH3 and CH4 heterodimers based on a mix of homodimeric interfaces.** The IMGT numbering is used. Naming convention: the domain type is stated in parentheses (CH3 or CH4) followed by the abbreviation of the engineered domain wherein position 88 was exchanged and then the abbreviation of the engineered domain wherein positions 85.1 and 86 were exchanged. Engineered domains are denoted by a two-letter abbreviation as follows: the 1st letter corresponds to the Ig class of position 88, and the 2nd letter corresponds to the Ig class of positions 85.1 and 86. Charged residues are colored in *red* (negative) or *blue* (positive). Hydrophobic interactions are shown as *gray lines*, and electrostatic interactions are shown as *dashed red lines*. Grafted residue numbers are in *yellow*.

**Figure 6.**
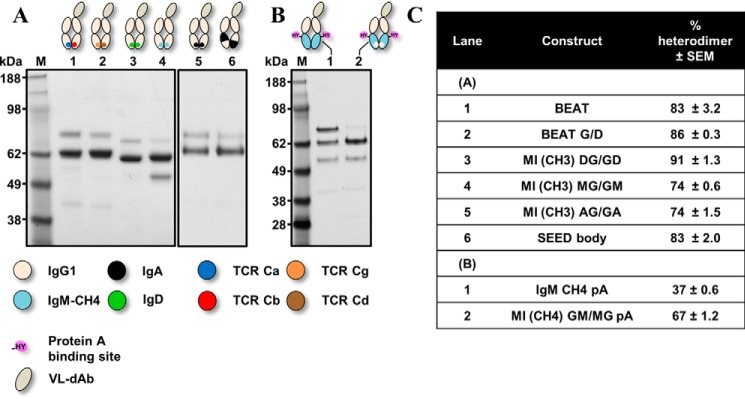
**SDS-PAGE analysis of the new CH3 and CH4 heterodimers based on a mix of homodimeric interfaces.** Engineered Fc-like proteins were transiently expressed, purified by Protein A chromatography, and analyzed by SDS-PAGE. *A,* BEAT Fc and BEAT G/D Fc *versus* Fc-like proteins with a CH3 heterodimer based on half-donor interfaces from IgA CH3, IgD CH3, or IgM CH4 grafted onto the IgG1 CH3 domain pair: MI (CH3) AG/GA, MI (CH3) DG/GD, and MI (CH3) MG/GM, respectively; the Fc-like protein based on the SEED technology is shown. *B,* MI (CH4) GM/MG pA corresponds to an Fc-like protein with a CH4 heterodimer based on grafting half of the IgG1 CH3 interface onto the IgM CH4 domain pair; the engineered CH4 heterodimer was also engineered to bind Protein A. The Fc-like protein IgM CH4 pA is used as a control. *C,* summary of heterodimer content.

Because of the inherent symmetry found in homodimeric interfaces, the four key sets of interface positions identified in heterodimers only translate into two sets. Hence, one of the two acceptor domains is designed with substitutions at positions 88 and 20 with supporting substitutions at positions 79, 81, and 90, whereas the other domain is substituted at positions 26, 85.1, and 86 with supporting substitutions at positions 3, 5, 84, and 84.2. New heterodimers based on mixed homodimeric interfaces are referred to as mixed interface (MI) heterodimers (Fig. 5 and supplemental Fig. S1). Using the methods described above, Fc-like proteins with mixed interface CH3 or CH4 heterodimers were built and tested as previously shown for our TCR grafts (Fig. 6).

Our mixed IgD/IgG1 CH3 heterodimer (MI (CH3) DG/GD) had the highest HD rate of the MI grafts, reaching 91% (Fig. 6*A*, *lane 3*). MI CH3 heterodimers based on IgA and IgM had HD rates above KiH but lower than SEED (Fig. 6*A*, *lanes 4* and *5*), whereas the MI CH4 heterodimer (MI (CH4) GM/MG pA) was identical to KiH (Fig. 6*B*, *lane 2*) but had a lower rate compared with BEAT CH4 pA. The SEED technology (14) is an HD technology wherein several β-strands and loops are exchanged between IgG1 CH3 and IgA CH3 to create a new CH3 heterodimer. As Trp 81 mediated few hydrophobic interactions within IgA CH3 as well as in our homology models of SEED (supplemental Fig. S4) and MI (CH3) AG/GA (Fig. 5), we back mutated this position for threonine to probe any differences between the two interfaces. HD assays with the back-mutated heterodimers showed that this position was important in SEED but had little impact on the MI (CH3) AG/GA heterodimer (supplemental Fig. S5, 20% *versus* 4% loss in HD rate, respectively), demonstrating that the two methods bring a different network of interactions. Our models did not show any differences between SEED and MI (CH3) AG/GA regarding the hydrophobic interactions mediated by Trp 81, but the involvement of core residues and/or additional interactions found in SEED outside of the IMGT positions used in our method cannot be excluded. In contrast, the model of our heterodimer showed three electrostatic contacts that were not found in the SEED model (Glu 13 (AG)/Arg 26 (GA), Lys 16 (AG)/Glu 3 (GA), and Arg 90 (AG)/Asp 84.4 (GA)).

### Rapid and scalable reformatting of antibody pairs into bsAbs

**Figure 7.**
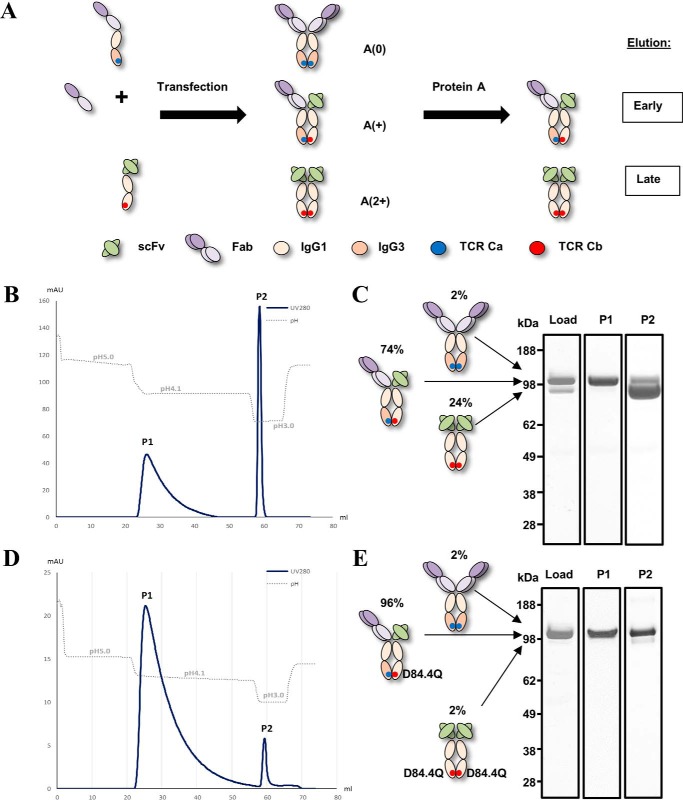
**Differential Protein A chromatography of BEAT antibodies.** *A,* schematics of asymmetric Protein A binding strategy for platform removal of trace amounts of homodimers. Upon transient transfection, the scFv × Fab BEAT antibody is assembled from three different chains (*middle species*); traces of two homodimeric contaminants can also be formed (*top* and *bottom species*) but are easily purified by Protein A chromatography; the Ig-like contaminant (*top species*) does not bind Protein A, whereas the scFv-Fc dimer contaminant (*bottom species*) binds more strongly than the bispecific antibody thereby allowing separation. *A*(*0*) means no Protein A-binding site; *A*(+) means one Protein A-binding site; and *A*(*2*+) means two Protein A-binding sites. *B* and *C,* BEAT 2/3. *B,* Protein A purification chromatogram. *C,* SDS-PAGE analysis of main peak fractions. *D* and *E,* BEAT 2/3 variant with the D84.4Q substitution. *D,* Protein A purification chromatogram. *E,* SDS-PAGE analysis of main peak fractions. Gels include a control lane (Load) corresponding to culture supernatant after Protein G purification that shows the hetero-to-homodimer ratio before loading on the Protein A column.

bsAbs were engineered using our original BEAT interface as this interface was well characterized at the time this work was performed. Lc mispairing was addressed by having two different antigen binding formats on each Hc: an scFv on one Hc and a Fab on the other Hc (scFv × Fab). To produce bsAbs free of homodimers, BEAT antibodies were further engineered to have asymmetric Protein A binding. A difference in Protein A binding between the two Fc chains was created by having one Fc chain originating from human IgG3, a natural IgG isotype that does not bind Protein A (Fig. 7*A*).

**Figure 8.**
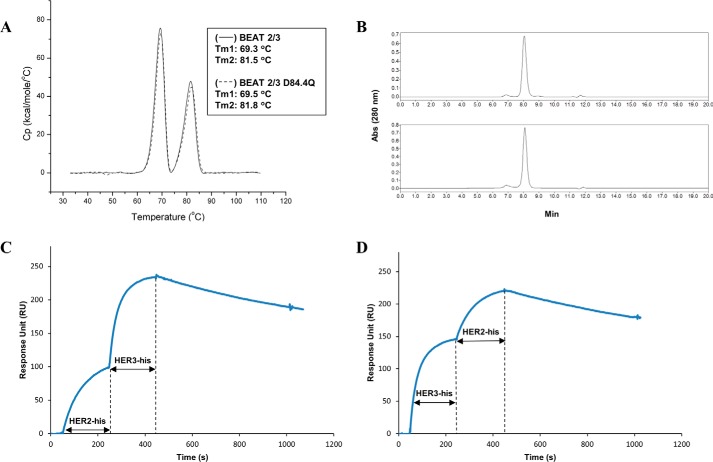
**Biochemical characterization of BEAT 2/3.** *A,* thermal stability by DSC. An overlay of the BEAT 2/3 without (*solid line*) and with the D84.4Q substitution (*dashed line*) is shown. The first transition corresponds to the melting of the scFv moiety and the BEAT Fc region (∼69.5 °C), and the second transition accounts for the melting of the Fab portion (∼81.5 °C). *B,* size-exclusion chromatography profiles of BEAT 2/3 and its D84.4Q variant: *top*, BEAT 2/3 (94% monomer); *bottom*, BEAT 2/3 variant with the D84.4Q substitution (92% monomer). *C* and *D,* co-binding of BEAT 2/3 to recombinant HER2 and HER3 extracellular domains monitored by SPR; the length of each antigen injection is indicated by a *double-headed arrow*.

In this study, dual targeting BEAT antibodies against the clinically relevant targets EGFR (epidermal growth factor receptor), HER2 (human epidermal growth factor receptor 2), and HER3 (human epidermal growth factor receptor 3) were prepared. Marketed antibodies against EGFR (Erbitux, Bristol-Myers Squibb, Merck KGaA, and Lilly) (20) and HER2 (Herceptin, Roche-Genentech) (21) were used as a source of variable domains. Our anti-HER3 variable domains were obtained from an anti-HER3 antibody in preclinical development (antibody U1-59) (22). Two target combinations were prepared and scaled-up: BEAT 1/3 (Erbitux (scFv) × anti-HER3 (Fab)) and BEAT 2/3 (Herceptin (scFv) × anti-HER3 (Fab)). The bsAbs were transiently expressed in HEK293-EBNA cells with yields between 10 and 25 mg/liter. HD levels in the scFv × Fab format were lower than those observed earlier with the monovalent dAb format (74%; Fig. 7, *B* and *C*). Homodimer contaminants were efficiently removed using Protein A chromatography based on two isocratic elution steps. Retrospectively, it was found that adding the D84.4Q substitution in chain (B) greatly improved HD of BEAT antibodies (96%; Fig. 7, *D* and *E*). BEAT antibodies exhibited good DSC and SEC profiles; for example, BEAT 2/3 displayed a thermogram with no transition below 69 °C, and introducing the D84.4Q substitution had no impact on its thermal stability (Fig. 8*A*). Additionally, the bsAb showed a single monomeric peak by SEC (Fig. 8*B*). BEAT 2/3 affinities for Fcγ receptors were comparable with that of Herceptin (IgG1) (supplemental Table S1). Improvements in affinities for FcγR2a, FcγR2b, and FcγR3a were observed for a BEAT 2/3 variant that included both a CH2 and a CH3 from IgG3 in one of its Fc chains. The D84.4Q substitution in BEAT 2/3 reduced affinities for FcγR1a, FcγR2a, and FcγR2b by 2-fold, whereas FcγR3a affinity was reduced by 25%, although still in the range of previously reported values for other IgG1 antibodies (23).

Human IgG3 immunoglobulins are known to have a shorter circulating half-life (24); however, using the IgG3 isotype in one of the two Fc chains (CH2-CH3 or CH3 from IgG3) had no impact in terms of FcRn binding, and measured affinities were in-line with reported values (supplemental Table S1) (25). Pharmacokinetics studies in rats showed that BEAT antibodies benefited from a prolonged half-life similar to that of previously reported Hc heterodimers (26, 27); for example, an elimination half-life (*t*_½_) of 164 h following i.v. injection of 10 mg/kg of BEAT 2/3 was observed (supplemental Table S2; note that antibody U1-59 has been reported to cross-react with rat HER3 (22)).

**Figure 9.**
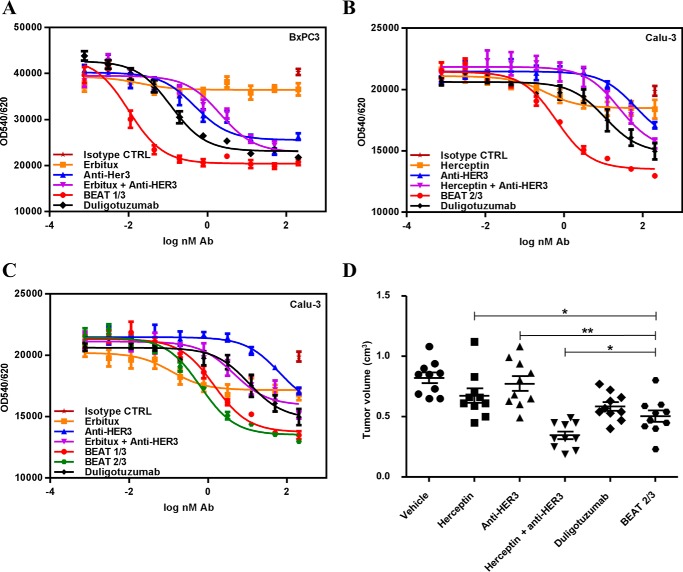
***In vitro* and *in vivo* efficacy of BEAT antibodies.** *A–C,* BEAT 1/3 and 2/3 inhibited tumor growth *in vitro* more potently compared with the monotherapies and their combinations. BxPC3 (*A*) and Calu-3 (*B* and *C*) cells were treated with antibodies or a combination of antibodies in a dose-dependent manner in the presence of heregulin. Cell proliferation was assessed after 72 h of treatment using Alamar Blue. Data are shown as mean ± S.E. plotted from one representative experiment. *D,* BEAT 2/3 inhibited tumor growth *in vivo* more potently compared with the monotherapies. Mice were treated every 3–4 days from day 12; tumor growth was monitored until day 40. Monotherapies and bispecific antibodies were dosed at 15 mg/kg; for combination therapies, each antibody was injected at 7.5 mg/kg. Data are presented as the tumor size (cm^3^) of each mouse at day 40 after xenograft; for each group, the mean ± S.E. is shown. Control (*CTRL*) indicates treatment with PBS. *Asterisks* denote statistically significant *p* values.

BEAT antibodies retained the respective binding affinities of their parental antibodies (20–22); for example, BEAT 2/3 was able to co-engage its targets (Fig. 8, *C* and *D*) with affinities of about 2–3 nm (supplemental Table S3). BEAT 1/3 and 2/3 induced antibody-dependent cellular cytotoxicity against A431 and SKBR3 cells with similar levels as that seen with Erbitux and Herceptin, respectively (data not shown). Both bsAbs were further tested at inhibiting the proliferation of different cancer cell lines (Fig. 9, *A–C*). Duligotuzumab, a bsAb directed against EGFR and HER3, which is currently in phase Ib for first-line treatment of recurrent/metastatic squamous cell carcinoma of the head and neck (28, 29), was used as a control. BEAT 1/3 and 2/3 were better at inhibiting BxPC3 (Fig. 9*A*) and Calu-3 cell proliferation (Fig. 9, *B* and *C*) over monotherapies, combination of parental antibodies, and duligotuzumab, respectively.

HER2/HER3 targeting antibodies may constitute a promising therapeutic strategy in cancer (30); hence, we decided to assess the *in vivo* antitumor efficacy of BEAT 2/3 in tumor xenograft experiments. Naive SCID beige mice were xenografted subcutaneously with the Calu-3 cell line and treated with BEAT 2/3, duligotuzumab, Herceptin, anti-HER3 antibody, and the combination therapy of Herceptin and anti-HER3 antibody. The mean of Calu-3 tumor growth at day 40 after xenograft was used to assess antitumor efficacy (Fig. 9*D* and supplemental Table S4). Although the combination therapy was the most efficacious treatment, BEAT 2/3 was as efficacious if not better than duligotuzumab in this model.

## Discussion

We aimed to design a complete Hc HD platform that would address the current bottlenecks and integrate into the standard manufacturing processes used for marketed monoclonal antibodies. To minimize misassembled Hc contaminants, we set out to establish a new HD technique that is more efficient than some of the previously described methods (6, 14). We engineered a technology based on biomimicry wherein the interface of a hetero- or a homodimer is grafted onto a recipient homodimer to create a new heterodimer. Natural heterodimers provide a complete interface (Fig. 1*A*), whereas only half-interfaces from homodimers are grafted to generate a new heterodimeric interface from two homodimeric ones (Fig. 1*B*). A successful exchange is based on the concept of substituting a residue in a recipient homodimer interface with the residue from a donor homo- or heterodimer interface that is found at the equivalent 3D position. Conveniently, 3D equivalent positions among Ig constant domain pairs are readily identified via the IMGT numbering system (17, 18) thereby providing a solution in terms of the amino acid choice that is available to create a new heterodimeric interface. Other HD technologies (6, 9, 11–14) do not provide for such a generic solution but rather a case-by-case approach wherein every new heterodimer is designed *de novo* without prior knowledge of the choice of substituting residues. Another important aspect was the intrinsic propensity of the two domains to readily heterodimerize, as many technologies often rely on an optimization of the ratio of the two Fc chains to increase HD levels in transient transfections (9, 13). Hence, we monitored the assembly of our heterodimer candidates in transient transfections using equal quantities of each engineered chain.

As a starting point, we designed Fc heterodimers based on engineered IgG1 CH3 or IgM CH4 domain pairs wherein HD was driven by an interface exchange of the homodimer protein-protein interface with either a complete heterodimeric interface (TCR Ca-Cb and TCR Cg-Cd) or half of a homodimeric interface (IgA CH3, IgD CH3, IgG1 CH3, and IgM CH4). These Fc-like proteins were fused to a single dAb (monovalent format) and transiently transfected at a 1:1 chain ratio.

Grafting the TCR Ca-Cb interface to engineer a new CH3 heterodimer (Fig. 2) was one of the most successful strategies, which after some optimization of the initial graft led to a near 95% in HD, well above the levels observed for the KiH (6) and SEED (14) technologies (Fig. 3). Upon solving the crystal structure of this initial graft, we observed the successful importation of key TCR contacts (Fig. 4) as well as the formation of new interactions not found in the parental interfaces.

Exchanging half-interfaces between homodimers (Fig. 5) was also successful at driving HD (Fig. 6). Interface exchange between IgG1 CH3 and IgD CH3 was the most successful of our half-interface grafts suggesting that exchanging half-interfaces between Ig homodimers can be as successful as SEED when considering the added benefit of introducing fewer substitutions with no insertions (supplemental Fig. S1). Interestingly, the majority of the MI heterodimers had a lower HD rate than BEATs suggesting that heterodimeric interface exchange is more efficient probably due to the fact that natural heterodimers provide a complete interface, which has been evolutionarily selected as heterodimeric.

Nonetheless, our method could be broadly implemented across the antibody Ig constant domain family, and we successfully built seven examples of new CH3 or CH4 heterodimers with six new heterodimers exhibiting HD levels above KiH. To our knowledge, CH4 heterodimers have not been previously reported and may prove useful in the design of new antibody therapeutics.

In the second part of our study, we selected our BEAT Fc heterodimer (original TCR Ca-Cb graft) to design bsAbs based on a good HD rate and the expression, stability, and putative low immunogenicity profile (supplemental Fig. S3). Regardless of the HD level achieved, we and others (7, 12, 31) found that Hc heterodimers do contain at least trace amounts of homodimer contaminants following scale-up, and we therefore decided to engineer our heterodimeric Fc for asymmetric Protein A binding by using a human IgG3 CH3 domain in one of the Fc chains, thereby enabling platform removal of any homodimeric species (Fig. 7*A*). Previous to our work, asymmetric Protein A-binding strategies for Hc homodimer removal have not been used in combination with a CH3 HD technology but have been successfully used by other groups as stand-alone techniques (15, 32, 33). However, without CH3 HD technology, bsAb recovery is poor (<50% of bispecific content at harvest). Finally, to address the Lc pairing issue inherent to Hc heterodimers, we used the scFv × Fab format. Aside from developing a common Lc, using this format is one of the most straightforward ways not only to bypass Lc mispairing but also to avoid additional side products, as well as enabling a rapid reformatting of two different parental antibodies in a bsAb. In our view, technologies that steer Lc pairing may not be sufficient to completely prevent mispairing (10, 16) and thus may require additional downstream purification steps when scaled up. One key benefit of the scFv × Fab format is its unique molecular weight signature that facilitates analytical analysis.

Proof-of-concept BEAT antibodies were designed to co-target different pairs of validated tumor antigens (EGFR/HER3 and HER2/HER3). HD rates in the scFv × Fab format were lower than the levels observed with the monovalent dAb format discussed earlier and usually reached 70–75% in transient transfections (Fig. 7, *B* and *C*); optimizing chain ratios, adding the Q3A substitution in chain (A) or different substitutions at position 90 in chain (B), helped to achieve levels of >75%. Retrospectively to our structural study and the making of our proof-of-concept bsAbs, we identified the D84.4Q substitution that was found to greatly improve HD in this format, most likely by disfavoring the formation of (B) chain homodimers. Adding the D84.4Q substitution to the original BEAT interface allowed HD levels to routinely reach between 85 and >95% at a 1:1:1 chain ratio (Fig. 7, *D* and *E*). The variability observed for the original BEAT interface and to a lesser extent for the BEAT D84.4Q interface also correlated with the nature of the parental antibodies and their expression levels and how well these antibodies converted into the scFv format; however, stably transfected CHO clones using the original interface consistently showed HD levels with a median of distribution of ∼80% with some clones reaching levels of >90–95% (34). The combination of good HD and platform removal of homodimer traces give robustness to our technology as it enables a clone selection based on a range of good properties (yield, stability, and glycosylation profile) rather than selecting purely on HD rate.

BEAT antibodies had IgG1-like affinities for Fc receptors (supplemental Table S1), good affinities for their target antigens (supplemental Table S3), good biophysical properties (Fig. 8), and benefited from a prolonged serum half-life in rodents (supplemental Table S2). The bsAbs exhibited superior *in vitro* efficacy over another anti-EGFR/HER3 bsAb benchmark (two-in-one antibody technology) (Fig. 9, *A–C*) (29). Importantly, our anti-HER2/HER3 BEAT antibody showed comparable efficacy to the two-in-one antibody in a Calu-3 xenograft model (Fig. 9*D* and supplemental Table S4), thereby validating our bispecific format for the design of future therapeutic bsAbs.

In summary, we have shown that interfaces between Ig constant domain pairs can be exchanged to design new heterodimers and that combining interface exchange, asymmetric Protein A binding, and the scFv × Fab format enabled the rapid reformatting and scale-up of any antibody pair into a bsAb.

## Experimental procedures

### Molecular biology

cDNA coding sequences of engineered CH3 and CH4 domains were designed *in silico* prior to being gene-synthesized at GENEART AG (Regensburg, Germany). The engineered Fc-like proteins based on CH4 or CH3 domains were engineered as follows: a first chain comprising a human IgG1 hinge, an IgG1 CH2 domain, and a first engineered CH3 or CH4 domain; and a second chain comprising a human VL domain, an IgG1 hinge, an IgG1 CH2 domain, and a second engineered CH3 or CH4 domain. Protein A binding in CH4 domains was engineered via the R115H and V116Y substitutions (IMGT numbering). PCR products were cloned into a modified pcDNA3.1 plasmid (Invitrogen AG, Basel, Switzerland) carrying oriP, which is the origin of plasmid replication of Epstein-Barr virus. For asymmetric Protein A binding, BEAT antibodies were engineered with a BEAT CH3 (A) domain originating from human IgG3; the BEAT 2/3 antibody used in the rat pharmacokinetic study was engineered with both CH2 and CH3 domains originating from human IgG3 in its BEAT Fc chain (A).

### Transient expression and purification

Recombinant Fc-like proteins were produced by co-transfection of a first engineered Fc-like chain and a second VL-Fc-like chain. BEAT antibodies were expressed by co-transfecting a first scFv-Fc-like heavy chain (scFv arm), a second engineered heavy chain (Fab arm), and the cognate light chain to the Fab arm heavy chain. Equal quantities of each engineered chain vector were co-transfected into suspension-adapted HEK293-EBNA cells (catalog no. ATCC-CRL-10852, LGC Standards, Teddington, UK) using polyethyleneimine. Cells were cultured for a period of 4–5 days before harvest. Cell-free culture supernatants were prepared by centrifugation followed by filtration. Fc-like proteins were purified by Protein A chromatography operated under gravity flow (CaptivA® Protein A resin, Repligen, Waltham, MA) before SDS-PAGE analysis (4–12% acrylamide). For BEAT antibodies, aliquots of cell-free supernatants were first purified by Protein G chromatography operated under gravity flow (Sepharose 4 Fast Flow; GE Healthcare Europe GmbH, Glattbrugg, Switzerland) to assess heterodimer content by SDS-PAGE before differential Protein A chromatography.

### Heterodimerization assay of Fc-like proteins

The proportions of heterodimer to homodimers in the Protein A-purified preparations were determined by scanning densitometry analysis of the non-reduced SDS-polyacrylamide gel bands (4–12%, Coomassie staining). Traces of the VL-Fc-like chain (half-molecule, 37.2 kDa) were found for most Fc-like proteins; hence bands below the 49-kDa marker were excluded from measurements. Relative ratios of the different gel bands were quantified using a FluorChem SP imaging system (Witec AG, Littau, Switzerland) following the manufacturer's protocol.

### BEAT Fc preparation, crystallization, and structure determination

DNA constructs for crystallization were prepared as follows. A cDNA coding the engineered CH3 domain of BEAT (A) (IgG3 isotype) and the engineered CH3 domain of BEAT (B) (IgG1 isotype) were synthesized. Using PCR assembly techniques, each chain had their respective engineered CH3 domain cDNA coding sequence fused downstream of a human IgG1 hinge (DKTHTCPPCP) and an IgG1 CH2 constant domain sequence (separately synthesized). A polyhistidine sequence was fused at the C terminus of the BEAT (A) chain (BEAT (A) His chain). Finally, the BEAT (A) His chain and BEAT (B) chain coding DNA sequences were ligated into independent vectors and co-expressed in HEK293-EBNA cells as described above. To ensure complete removal of homodimer contaminants (which were not identifiable by SDS-PAGE due to the almost identical molecular weights of both chains), the BEAT Fc was purified in a two-step procedure. In a first step, the filtered cell culture supernatant was applied to a Protein A-Sepharose column (GE Healthcare Europe GmbH). Because the BEAT (A) CH3 domain was of the IgG3 isotype, no potential BEAT (A) His chain homodimers bound Protein A. After loading, the column was washed with phosphate buffer (20 mm Na_2_HPO_4_, pH 8, + 0.15–1 m NaCl). Elution was achieved with 0.1 m glycine, pH 3, followed by immediate neutralization of the sample with 10% v/v of 1 m Tris-HCl, pH 8. Eluted fractions were further purified in a second step via Ni^2+^-nitrilotriacetic acid resin (Qiagen GmbH, Hombrechtikon, Switzerland) according to the manufacturer's protocol, a step that eliminated any potential BEAT (B) homodimers as these do not carry a polyhistidine tag sequence. The purified BEAT Fc was dialyzed against PBS, pH 7.4. Crystallization trials and structure determination were performed at Crelux GmbH (Martinsried, Germany). In brief, crystals of BEAT Fc were obtained using hanging drop vapor diffusion setups after reductive methylation of the protein (Hampton Research Reductive Alkylation Kit, catalog no. HR2-434, Hampton Research, Aliso Viejo, CA). 0.8 μl of BEAT Fc solution (10.2 mg/ml in 10 mm Tris-HCl, 0.1 m NaCl, 1 mm EDTA, pH 8) was mixed with 0.8 μl of reservoir solution (27% (w/v) PEG1500) and equilibrated at 20 °C over 0.2 ml of reservoir solution. Well diffracting crystals appeared within 1 day and grew over 3–4 days to full size. A complete data set of a BEAT Fc crystal was collected at the Diamond Synchrotron Radiation Source, Beamline i04, Didcot, UK. Molecular replacement was performed using one chain of a published human IgG1 Fc structure (PDB accession code 1H3T) as a search model (35). Several rounds of alternating manual rebuilding and refinement with REFMAC5 (36) resulted in the final model. Data deposition: the atomic coordinates of the BEAT Fc structure have been deposited with the Protein Data Bank under code 5M3V.

### Antibody modeling

Homology modeling was performed using the YASARA software (37). Interdomain interactions were analyzed using the on-line PIC server (http://pic.mbu.iisc.ernet.in/)^5^ (38). The interdomain interactions for IgG1 CH3 were calculated from PDB code 1H3T. Interactions in TCR Ca-Cb were analyzed from PDB codes 1KGC and 2CDF. Interactions in TCR Cg-Cd were analyzed from PDB code 1HXM. The BEAT CH3 heterodimer was first modeled from PDB code 1H3T, and interdomain interactions were analyzed for engineering; upon structure determination of the BEAT Fc, interdomain interactions were analyzed and reported herein. A homology model for BEAT min was calculated based on the solved structure of the BEAT Fc. For BEAT G/D, a homology model was built based on PDB code 1H3T, and the interdomain interactions were analyzed. As there is no solved crystal structure available for IgM, a homology model of IgM CH4 was built based on IgG1 CH3 (PDB code 1H3T). A homology model for BEAT CH4 was then built based on the homology model of IgM CH4. Homology models for MI heterodimers were built based on IgG1 CH3 (PDB code 1H3T). Interdomain interactions in IgA CH3 were calculated from PDB code 1OW0. A homology model of the SEED body CH3 heterodimer was built based on PDB code 1H3T. As there is no solved crystal structure available for IgD, a homology model for IgD CH3 was built based on PDB code 1H3T.

### Differential Protein A chromatography of BEAT antibodies

Cell-free supernatants were loaded onto a 1-ml HiTrap^TM^ MabSelect SuRe^TM^ Protein A column pre-equilibrated in 0.2 m citrate/phosphate buffer, pH 6, and operated on an ÄKTA^TM^ purifier chromatography system (both from GE Healthcare Europe GmbH) at a flow rate of 1 ml/min. Running buffer was 0.2 m citrate/phosphate buffer, pH 6. Wash buffer was 0.2 m citrate/phosphate buffer, pH 5. Heterodimer elution was performed using 20 mm sodium acetate buffer, pH 4.1, although homodimeric species were unbound (IgG-like homodimers) or eluted with 0.1 m glycine, pH 3 (scFv-Fc homodimers). Elution was followed by absorbance reading at 280 nm; relevant fractions were pooled and neutralized with 0.1 volume of 1 m Tris-HCl, pH 8. Fractions were analyzed under non-reduced conditions by SDS-PAGE.

### SPR analysis of BEAT antibodies

Experiments were performed on a Biacore 2000 or Biacore T200 instrument (GE Healthcare Europe GmbH) at room temperature. Data fitting was performed using the BIAevaluation software version 4.1 or the Biacore T200 Evaluation Software version 3.0 (both from GE Healthcare Europe GmbH).

### Binding affinities for EGFR, HER2, and HER3 extracellular domains

Protein A (Thermo Fisher Scientific, LuBioScience GmbH, Lucerne, Switzerland) or Protein G (Thermo Fisher Scientific) were coupled to a CM5 sensor chip (GE Healthcare Europe GmbH) using an amine coupling kit (GE Healthcare Europe GmbH). Before each antigen injection, BEAT antibodies were captured to reach about 200 response units. EGFR, HER3, and HER2 (extracellular domains produced in-house as polyhistidine-tagged proteins) were injected at 3–100 nm. Regeneration was performed with 0.1 m glycine, pH 1.5. Data fitting was performed using the 1:1 Langmuir binding model.

### Antigen co-binding experiments

BEAT antibodies were captured on a Protein G-coupled CM5 sensor chip to reach ∼200 response units. After response stabilization, relevant antigens (EGFR, HER2, or HER3) were sequentially injected at 200 nm for 4 min.

### Binding affinities for human Fc receptors

Human Fc receptor extracellular domains were cloned, expressed, and purified in-house. *K_D_* values for FcγR2a (UniProt P12318, variant H167), FcγR2b (UniProt P31994), FcγR3a (UniProt P08637, variant V176), and FcRn (UniProt P55899 and P61769) were measured via direct covalent coupling of the antibody onto a CM5 sensor chip. Dissociation was monitored for 2 min (FcγR2a, FcγR2b, and FcRn) or 12 min (FcγR3a). No regeneration step was needed for FcγR2a, FcγR2b, and FcγR3a as complete dissociation was observed. For FcRn measurements, regeneration was performed by injecting 2 × 10 μl of HBS-EP+, pH 7.4 (GE Healthcare Europe GmbH). All data were processed using the steady-state fitting model. HBS-EP+ buffer was used as running buffer; for FcRn measurements, HBS-EP+ buffer was adjusted to pH 6. Affinities for FcγR1a (UniProt P12314) were measured via Protein A capture. Experimental data for FcγR1a were processed using a 1:1 Langmuir model with the *R*_max_ value set to local in all fits. After each binding event, the surface was regenerated with 10 mm glycine buffer, pH 1.5.

### Differential scanning calorimetry

Measurements were carried out on a MicroCal VP-Capillary differential scanning calorimeter (Malvern Instruments Ltd., Malvern, UK). The cell volume was 0.128 ml, the heating rate was 1 °C/min, and the excess pressure was kept at 64 p.s.i. All protein samples were tested at a concentration of 1–0.5 mg/ml in PBS, pH 7.4. The partial molar heat capacities and melting curves were analyzed using standard procedures. Thermograms were baseline-corrected, and concentration was normalized before being further analyzed using a non-two-state model in the software Origin version 7.0 (supplied by Malvern Instruments Ltd).

### Size-exclusion chromatography

Analytical SEC was performed using a Tosoh Bioscience TSKgel G3000SWxl column (catalog no. 08541, Tosoh Bioscience AG, Lucerne, Switzerland) at room temperature with 0.1 m sodium phosphate buffer, 0.15 m sodium chloride, pH 6.8, as eluent at 1 ml/min flow rate, on a Waters Alliance 2695 HPLC system with a Waters 2998 PDA detector (Waters AG, Baden-Dättwil, Switzerland), monitoring at 214 and 280 nm.

### PK study

Pharmacokinetics analyses were conducted in female Sprague-Dawley rats. Each group contained four rats. Rats received 10 mg/kg BEAT 2/3 by intravenous bolus injection. Blood samples were collected at 0.25, 1, and 6 h and at 1, 2, 4, 5, 7, 10, 14, 21, 28, and 42 days post-injection. Serum levels of BEAT 2/3 were determined by sandwich ELISA. HER2 antigen (extracellular portion of HER2 fused to a human Fc and produced in-house) was coated onto 96-well ELISA plates at a concentration of 2 μg/ml and incubated overnight at 4 °C. After the plates were blocked with BSA, serum samples, reference standards (11 serial dilutions), and quality control samples were added to the plate and incubated for 1 h at room temperature. After washing to remove unbound antibody, peroxidase-conjugated goat anti-human IgG_F(ab′)_2_ fragment-specific detection antibody (Jackson ImmunoResearch, distributor: MILAN ANALYTICA AG, Rheinfelden, Switzerland, catalog no. 109-035-006) was added and developed by standard colorimetric tetramethylbenzidine substrate (Pierce-Thermo Fisher Scientific-Perbio Science S.A., Lausanne, Switzerland) according to the manufacturer's recommendations. Absorbance values at 450 nm were recorded on a plate reader, and the concentrations of antibody in serum samples were calculated using the reference standard curve generated in the sample plate utilizing four parametric regression models. The pharmacokinetics parameters were evaluated by non-compartment analysis using WinNonlin^TM^ version 5.3 (Pharsight Corp., Mountain View, CA).

### Reference antibodies

Analogue antibodies were cloned and expressed according to previously published variable domain sequences as follows: Herceptin (21), Erbitux (20), anti-HER3 (22), and duligotuzumab (39).

### Cancer cell lines

Calu-3 (catalog no. ATCC-HTB-55) and BxPC3 (catalog no. ATCC-CRL-1687) cell lines were purchased from American Type Cell Culture (ATCC-LGL standards, Teddington, UK). Calu-3 and BxPC3 cells were maintained in RPMI 1640 medium supplemented with 10% fetal calf serum (FCS) and 1% GlutaMAX/penicillin/streptomycin.

### Cell proliferation assays

Calu-3 (10,000 cells/well) and BxPC3 (8000 cells/well) cells were seeded in 96-well plates. The following day, cells were treated with antibodies or combinations of antibodies diluted in medium containing 1% FCS. For combination treatments, equal concentrations of antibodies were mixed to reach a total antibody concentration that was equal to the comparable single treatment. 3 nm heregulin (R&D Systems, Abingdon, UK, catalog no. 396-HB) was added after 1 h of incubation with antibodies or combinations. Alamar Blue (AbD Serotec, Düsseldorf, Germany) was added to the wells after 72 h, and the cells were incubated up to 24 h, before fluorescence was read on a Biotek Synergy 2 plate reader (BioTek Instruments GmbH, Luzerne, Switzerland) at an excitation wavelength of 540 nm and an emission wavelength of 620 nm.

### Xenograft studies

Xenograft studies were performed at Accelera Srl (Nerviano, Milano, Italy). *In vivo* experiments were performed in female 5-week-old naive SCID beige mice (Charles River Laboratories, Calco, Lecco, Italy). All experiments were done according to the Italian Animal Protection Law with authorization from the veterinary authorities. Calu-3 cells were injected subcutaneously (5 × 10^6^ cells) in the right flank of female naive SCID beige mice. When tumors had reached a volume of 100 to 300 mm^3^, mice were randomized and assigned to treatment groups, with a target of 10 mice per group. When treatment started, the mean tumor volume was ∼190 mm^3^ on day 12, and subsequently tumor growth was monitored every 3–4 days until day 40. Multiple-dose studies consisted of 5 weeks of treatment with the first dose on the day of randomization (intraperitoneal injections on day 15, 19, 22, 26, 29, 33, and 36). Tumor growth was determined by external caliper measurements, and tumor volumes were calculated using a standard hemi-ellipsoid formula (tumor volume (mm^3^) = 0.5 × length × (width)^2^). Monotherapies and bispecific antibodies were dosed at 15 mg/kg; for combination therapies, each antibody was injected at 7.5 mg/kg. For all *in vivo* results, data were analyzed using the Graphpad Prism 5 software (GraphPad Software Inc., La Jolla, CA). Data were analyzed for statistical significance using the one-way analysis of variance followed by a Dunnett's post hoc test for multiple comparisons or the Mann Whitney test for pairwise comparisons. *p* values of less than 0.05 were regarded as statistically significant.

## Author contributions

D. S., C. S., P. W., and S. B. designed research; D. S., C. S., R. O., E. S., P. W., F. B., T. M., and S. G. performed research; D. S., C. S., E. S., P. W., T. M., S. G., and S. B. analyzed data; and C. S. and S. B. wrote the paper.

## Supplementary Material

Supplemental Data

## Footnotes

5 Please note that the JBC is not responsible for the long-term archiving and maintenance of this site or any other third party hosted site.

## Footnotes

4 The abbreviations used are:

bsAb: bispecific antibody
EGFR: epidermal growth factor receptor
dAb: domain antibody
DSC: differential scanning calorimetry
SEC: size-exclusion chromatography
SPR: surface plasmon resonance
PDB: Protein Data Bank
TCR: T-cell receptor
Hc: heavy chain
KiH: knobs-into-holes
MI: mixed interface
HD: heterodimerization
BEAT: Bispecific Engagement by Antibodies based on the T-cell receptor
Lc: light chain
IMGT: International ImMunoGeneTics Information System.
