# Supplementary material for: Immunoglobulin domain interface exchange as a platform technology for the generation of Fc heterodimers and bispecific antibodies
Source: J Biol Chem. 2017 Apr 27;292(23):9745–59. doi: 10.1074/jbc.M117.782433 (PMC5465497; doi:10.1074/jbc.M117.782433)
Supplement: Supplemental Data [file supp_292_23_9745__index.html]

Immunoglobulin domain interface exchange as a platform technology for the generation of Fc heterodimers and bispecific antibodies — Immunoglobulin domain interface exchange as a platform technology for the generation of Fc heterodimers and bispecific antibodies — Interface exchange for bispecific antibody generation — Supplemental Data 

# Immunoglobulin domain interface exchange as a platform technology for the generation of Fc heterodimers and bispecific antibodies

## Supplemental Data

- Revised supplemental data (.pdf, 1.9 MB) - Revised supplemental data
