# Supplementary material for: Immunoglobulin domain interface exchange as a platform technology for the generation of Fc heterodimers and bispecific antibodies
Source: J Biol Chem. 2017 Apr 27;292(23):9745–59. doi: 10.1074/jbc.M117.782433 (PMC5465497; doi:10.1074/jbc.M117.782433)
Supplement: Supplemental Data [file 10.1074_M117.782433_jbc.M117.782433-1.pdf]

Supplemental Data for

Immunoglobulin Domain Interface Exchange as a Platform Technology for the Generation of Fc Heterodimers and Bispecific Antibodies

Darko Skegro[1,#], Cian Stutz[1], Romain Ollier[1], Emelie Svensson[1], Paul Wassmann[1,#], Florence Bourquin[1,$], Thierry Monney[1], Sunitha Gn[2], and Stanislas Blein[1*]

From the [1]Department of Antibody Engineering, Biologics Research, Glenmark Pharmaceuticals S.A., Chemin de la Combeta 5, 2300 La Chaux-de-Fonds, Switzerland; the [2]Department of Drug Metabolism and Pharmacokinetics, Glenmark Pharmaceuticals Limited, Glenmark Research Centre, Plot no.A-607, T.T.C. Industrial Area, MIDC, Mahape, Navi Mumbai-400 709, India.

[*]To whom correspondence should be addressed: E-mail: stanislas.blein@glenmarkpharma.com, Tel: +41 32 729 35 50.

Supplemental method

BEAT Fc preparation for DSC analysis–To assess the intrinsic stability of the BEAT Fc, a BEAT Fc chain (B) with a C-terminal HA tag but without VL domain was co-expressed in HEK293-EBNA cells with a BEAT Fc chain (A) fused to a C-terminal poly-histidine tag. The resulting Fc heterodimer was purified using a two-step affinity purification strategy; in a first step, the filtered cell-culture supernatant was purified via anti-HA affinity chromatography (Roche Diagnostics AG, Rotkreuz, Switzerland), and eluted fractions were further purified in a second step by Ni^{2+}-NTA chromatography (Qiagen GmbH, Hombrechtikon, Switzerland). Both chromatographic steps were run according to the resin manufacturer's protocol. The BEAT Fc material eluted from the final purification step was buffer exchanged into PBS before DSC analysis.

FIGURE S1. Amino acid sequences of parental and engineered CH3 and CH4 domains; previously described CH3 heterodimer sequences from the KiH and SEED technologies are included. Both the EU and IMGT numberings are shown for CH3 domains; for CH4 domains only the IMGT numbering is shown.

FIGURE S2. Schematic diagrams depicting the interfaces of BEAT min, IgM CH4, and BEAT CH4. The IMGT numbering is used. Charged residues are colored in red (negative) or blue (positive). Hydrophobic interactions are in grey lines, electrostatic interactions in dashed red lines. Grafted residue numbers are in yellow.

FIGURE S3. BEAT Fc. (A) BEAT Fc thermal unfolding by DSC. The single transition corresponds to the melting of the CH2 and engineered CH3 domains. (B) Solvent accessible surface area of the BEAT Fc highlighting TCR derived residues. TCR Ca derived residues are colored blue and TCR Cb derived residues are colored red.

FIGURE S4. Schematic diagrams depicting the interfaces of IgA CH3, IgD CH3, and SEED. The IMGT numbering is used. Charged residues are colored in red (negative) or blue (positive). Hydrophobic interactions are in grey lines, electrostatic interactions in dashed red lines. Grafted residue numbers are in yellow.

FIGURE S5. Impact of the W81T mutation in MI (CH3) AG/GA vs. SEED. (A) Fc-like proteins were purified by Protein A chromatography and monitored by SDS-PAGE. (B) Summary of heterodimer content.

TABLE S1. SPR binding affinities of BEAT 2/3 and variants thereof for human Fc receptors. Average KD values ± SD based on three or more replicate measurements are shown. KD values are reported as follows: FcγR1a = pM, FcγR2a/FcγR2b = μM; FcγR3a/FcRn= nM. For each measurement, the Rmax and χ2 values were used to evaluate the quality of the fit between the experimental data and the binding model; average Rmax and χ2 values ± SD based on three or more replicates for the same antibody-Fc receptor pair are shown; Rmax unit is RU and χ2 unit is RU^2. (a) BEAT 2/3 antibody used in cell assays and mouse xenograft studies. (b) BEAT 2/3 used in the rat pharmacokinetic study. The isotype of the different Ig constant domains within each BEAT Fc chain is indicated.

TABLE S2. Summary of the PK parameters in female Sprague-Dawley rats following iv bolus injection of the BEAT 2/3 antibody at 10 mg/kg. Abbreviations: $AUC_{0\text{-inf}}$ = area under the serum concentration-time curve from time zero to infinity; $AUC_{0\text{-tlast}}$ = area under the serum concentration-time curve from time zero to time point of last concentration measured; CL = clearance; C_{max} = maximum serum concentration; IV = intravenous; PK = pharmacokinetic; SD = standard deviation; $t_{½}$ = elimination half-life; T_{max} = time to reach maximum serum concentration following drug administration; Vz = apparent volume of distribution based on terminal phase; MRT_{inf} = mean residence time extrapolated to infinity. (*) median (min-max).

TABLE S3. SPR binding affinities of BEAT antibodies for EGFR, HER2, and HER3 extracellular domains. Average KD values ± SD based on two or more replicate measurements are shown. KD values are reported in nM. For each measurement, the Rmax and χ2 values were used to evaluate the quality of the fit between the experimental data and the binding model; average Rmax and χ2 values ± SD based on two or more replicates for the same antibody-antigen pair are shown; Rmax unit is RU and χ2 unit is RU^2. The isotype of the different Ig constant domains within each BEAT Fc chain is indicated. (n.a.) means non-applicable.

TABLE S4. Statistical analysis of Calu-3 xenograft studies. Method: one-way analysis of variance (ANOVA) followed by a Dunnett's post hoc test for multiple comparisons or Mann Whitney test for pairwise comparisons. P values of less than 0.05 were regarded as statistically significant. Asterisks denote statistically significant P values. (n.s.) means non-significant.

FIGURE S1

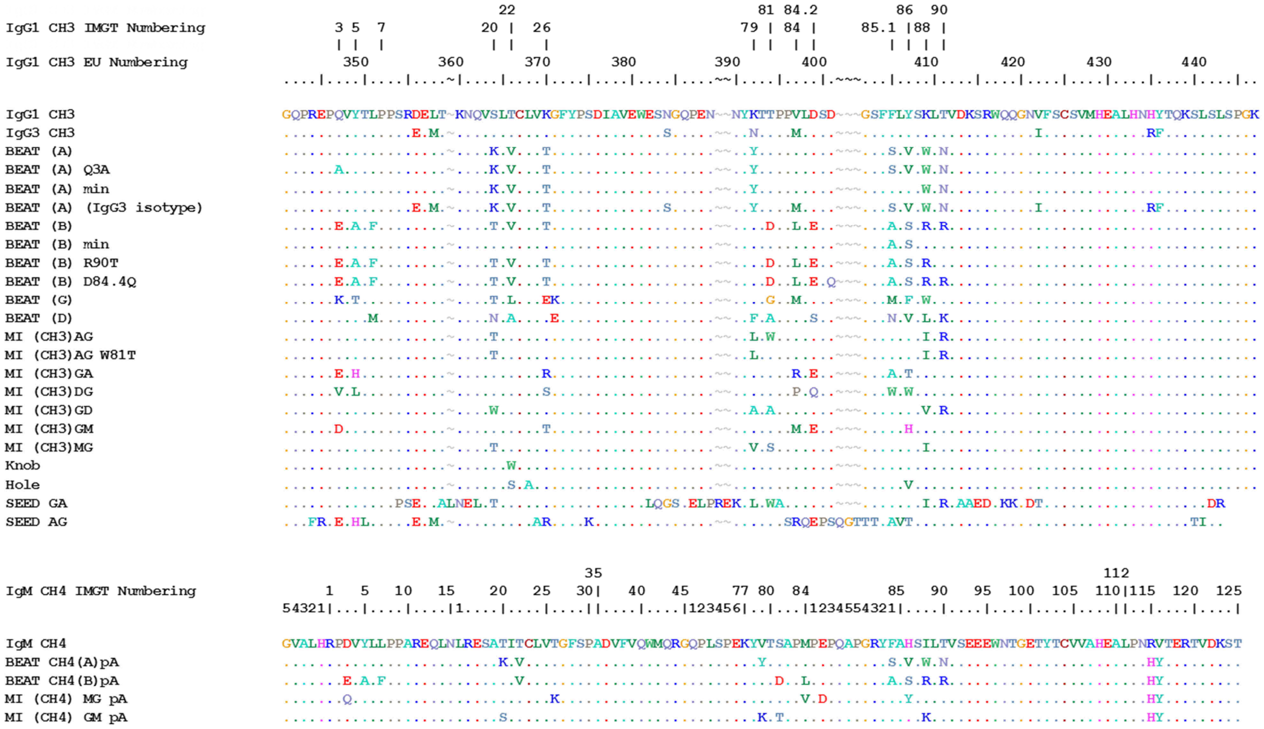
IgG1 CH3 IMGT Numbering
IgG1 CH3 EU Numbering
IgG1 CH3
GQPREPQVYTLPPSRDELT~KNQVSLTCLVKGFYPSDIAVEWESNGQPEN~~NYKTTPPVLDSD~~~GSFFLYSKLTVDKSRWQQGNVFSCSVMHEALHNHYTQKSLSLSPGK
IgG3 CH3
BEAT (A)
BEAT (A) Q3A
BEAT (A) min
BEAT (A) (IgG3 isotype)
BEAT (B)
BEAT (B) min
BEAT (B) R90T
BEAT (B) D84.4Q
BEAT (G)
BEAT (D)
MI (CH3)AG
MI (CH3)AG W81T
MI (CH3)GA
MI (CH3)DG
MI (CH3)GD
MI (CH3)GM
MI (CH3)MG
Knob
Hole
SEED GA
SEED AG
IgM CH4 IMGT Numbering
IgM CH4
GVALHRPDVYLLPPAREQLNLRESATITCLVTGFSPADVFVQWMQRGQPLSPEKYVTSAPMPEPQAPGRYFAHSILTVSEEEWNTGETYTCVVAHEALPNRVTERTVDKST
BEAT CH4(A)pA
BEAT CH4(B)pA
MI (CH4) MG pA
MI (CH4) GM pA

FIGURE S2

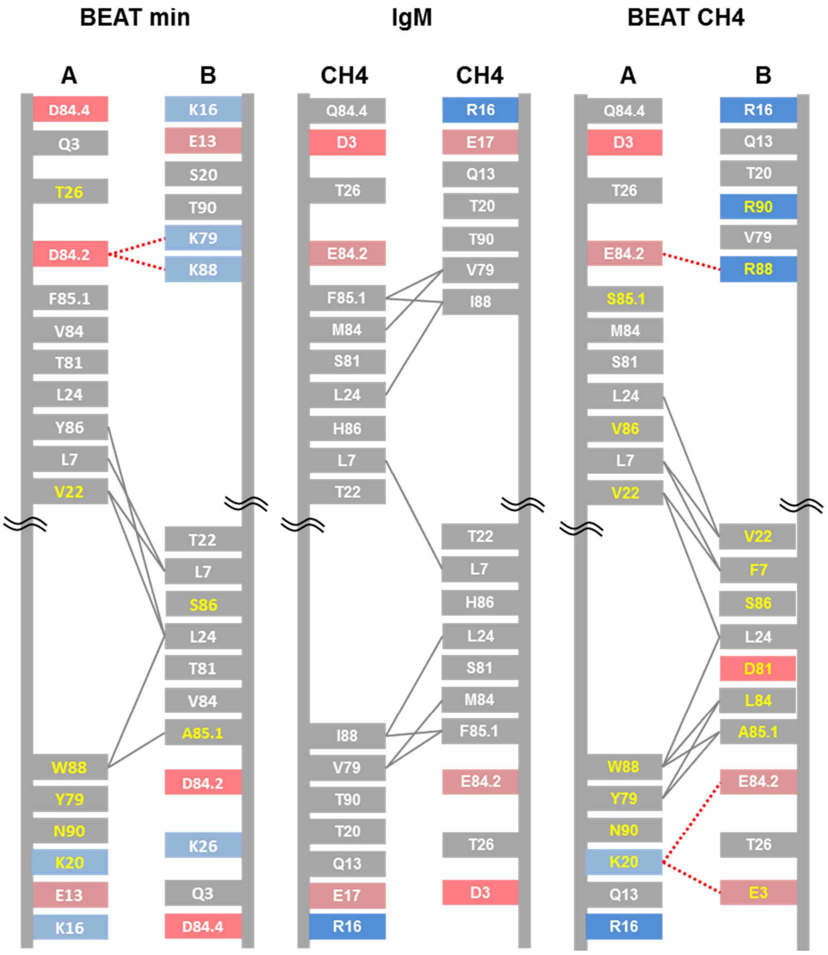

BEAT min
IgM
BEAT CH4
A
B
CH4
CH4
A
B
D84.4
Q3
T26
D84.2
F85.1
V84
T81
L24
Y86
L7
V22
W88
Y79
N90
K20
E13
K16
K16
E13
S20
T90
K79
K88
T22
L7
S86
L24
T81
V84
A85.1
D84.2
K26
Q3
D84.4
Q84.4
D3
T26
E84.2
F85.1
M84
S81
L24
H86
L7
T22
I88
V79
T90
T20
Q13
E17
R16
R16
E17
Q13
T20
T90
V79
I88
T22
L7
H86
L24
S81
M84
F85.1
E84.2
T26
D3
Q84.4
D3
T26
E84.2
S85.1
M84
S81
L24
V86
L7
V22
W88
Y79
N90
K20
Q13
R16
R16
Q13
T20
R90
V79
R88
V22
F7
S86
L24
D81
L84
A85.1
E84.2
T26
E3

FIGURE S3

A

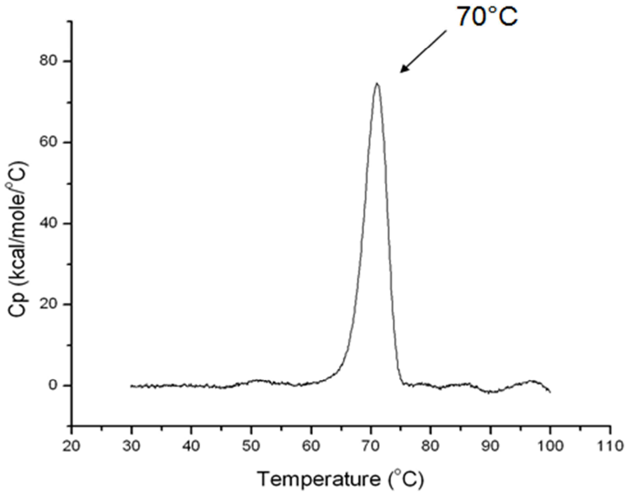

70°C
Cp (kcal/mole/°C)
80
60
40
20
0
20
30
40
50
60
70
80
90
100
110
Temperature (°C)

B

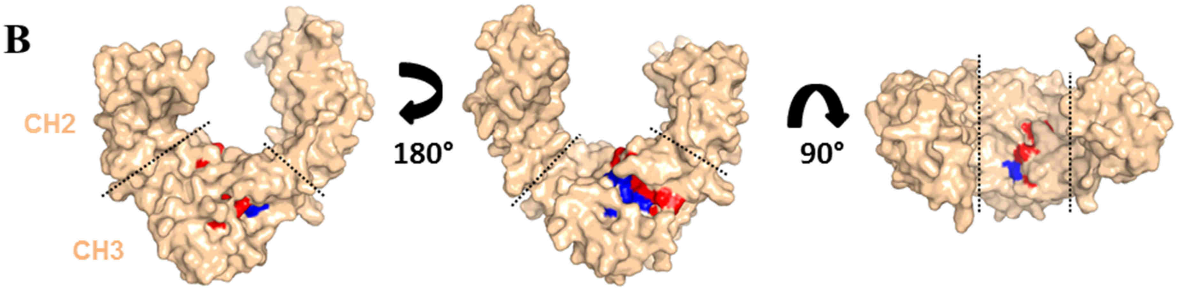

CH2
CH3
180°
90°

FIGURE S4

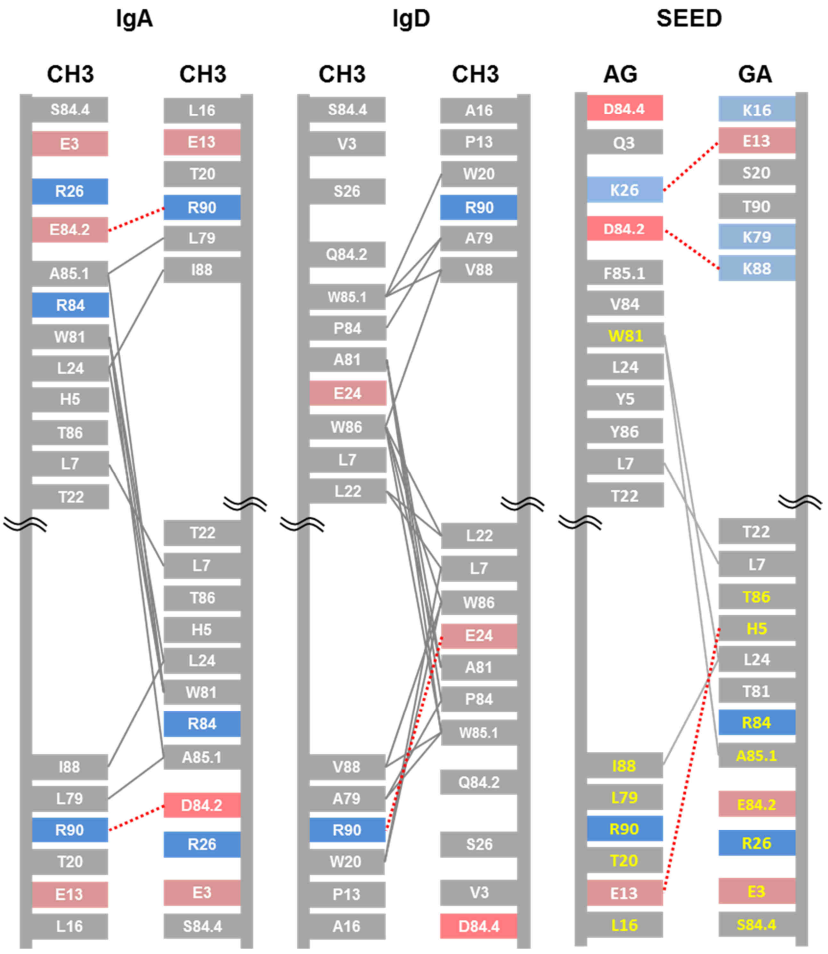

IgA
IgD
SEED
CH3
CH3
CH3
CH3
AG
GA
S84.4
E3
R26
E84.2
A85.1
R84
W81
L24
H5
T86
L7
T22
I88
L79
R90
T20
E13
L16
L16
E13
T20
R90
L79
I88
T22
L7
T86
H5
L24
W81
R84
A85.1
D84.2
R26
E3
S84.4
S84.4
V3
S26
Q84.2
W85.1
P84
A81
E24
W86
L7
L22
V88
A79
R90
W20
P13
A16
A16
P13
W20
R90
A79
V88
L22
L7
W86
E24
A81
P84
W85.1
Q84.2
S26
V3
D84.4
D84.4
Q3
K26
D84.2
F85.1
V84
W81
L24
Y5
Y86
L7
T22
I88
L79
R90
T20
E13
L16
K16
E13
S20
T90
K79
K88
T22
L7
T86
H5
L24
T81
R84
A85.1
E84.2
R26
E3
S84.4

FIGURE S5

A

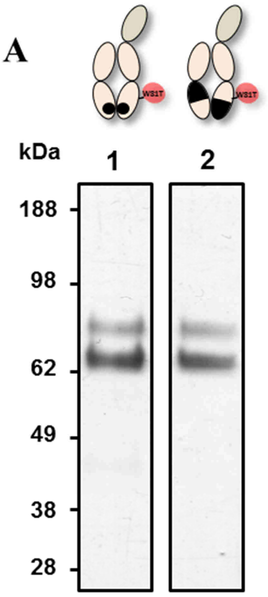

B

Lane	Construct	% heterodimer ± SEM
1	MI (CH3) AG/GA W81T	70 ± 0.7
2	SEED W81T	63 ± 1.9

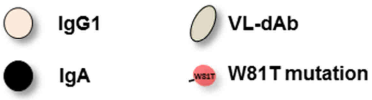

TABLE S1

Antibody		FcγR1a	FcγR2a	FcγR2b	FcγR3a	FcRn
BEAT 2/3[a]	KD	135 ± 14	2.12 ± 0.01	3.64 ± 0.05	206 ± 0.5	708 ±12.2
(A): CH2 γ1 - BEAT CH3 (A) γ3	Rmax	116 ± 30	122 ± 1	117 ± 1	136 ± 1	506 ± 4
(B): CH2 γ1 - BEAT CH3 (B) γ1	$\chi 2$	0.4 ± 0.17	6.5 ± 0.61	3.57 ± 0.17	23.8 ± 0.15	10.7 ± 0.97
BEAT 2/3[b]	KD	137 ± 12	1.49 ± 0.01	2.21 ± 0.01	105 ± 0.7	543 ± 10.5
A): CH2 γ3 - BEAT CH3 (A) γ3	Rmax	133 ± 55	120 ± 1	129 ± 1	161 ± 1	655 ± 19
(B): CH2 γ1 - BEAT CH3 (B) γ1	$\chi 2$	0.68 ± 0.47	8.75 ± 0.23	7.13 ± 0.28	22.33 ± 0.6	2.62 ± 0.98
BEAT 2/3	KD	298 ± 13	3.91 ± 0.03	7.2 ± 0.11	347 ± 5.1	686 ± 17.4
(A): CH2 γ1 - BEAT CH3 (A) γ3	Rmax	108 ± 1	107 ± 1	97 ± 1	116 ± 1	461 ± 5
(B): CH2 γ1 - BEAT CH3 (B) γ1 D84.4Q	$\chi 2$	0.32 ± 0.08	3.41 ± 0.13	1.08 ± 0.09	14.47 ± 0.5	1.92 ± 0.91
BEAT 2/3	KD	317 ± 11.5	2.36 ± 0.2	5.27 ± 0.5	238 ± 4.0	577 ± 13.1
(A): CH2 γ3 - BEAT CH3 (A) γ3	Rmax	110 ± 1	110 ± 2	103 ± 2	124 ± 1	510 ± 6
(B): CH2 γ1 - BEAT CH3 (B) γ1 D84.4Q	$\chi 2$	0.36 ± 0.01	4.02 ± 2.32	3.98 ± 2.22	14.17 ± 0.76	1.42 ± 1.43
Herceptin IgG	KD	138 ± 12	2 ± 0.2	4.27 ± 0.1	267 ± 3.9	622 ± 56.5
	Rmax	92 ± 2	61 ± 6	61 ± 4	78 ± 11	389 ± 53
	$\chi 2$	0.31 ± 0.19	2.47 ± 1.55	1.07 ± 1.14	10.43 ± 5.61	8.45 ± 5.79

TABLE S2

Mean PK Parameters	Units	Mean (SD)
		Female Sprague Dawley Rat (10 mg/kg; IV)
C_{max}	(µg/ml)	276 (29.72)
$AUC_{0\text{-}tlast}$	(h.µg/ml)	12204 (4290.39)
$AUC_{0\text{-}inf}$	(h.µg/ml)	12277 (4359.82)
$*T_{max}$	(h)	0.25 (0.25-0.25)
$t_{1/2}$	(h)	164 (55.39)
Vz	(ml/kg)	215 (121.14)
CL	(ml/min/kg)	0.015 (0.006)
MRT_{inf}	(h)	112 (30.37)

TABLE S3

Antibody		EGFR	HER2	HER3
BEAT 1/3	KD	0.3 ± 0.01	n.a.	2.5 ± 0.1
(A): CH2 γ1 - BEAT CH3 (A) γ3	Rmax	99 ± 1	n.a.	124 ± 7
(B): CH2 γ1 - BEAT CH3 (B) γ1	χ2	0.84 ± 0.23	n.a.	0.8 ± 0.12
BEAT 2/3	KD	n.a.	2.9 ± 0.9	2 ± 0.1
(A): CH2 γ1 - BEAT CH3 (A) γ3	Rmax	n.a.	128 ± 3	163 ± 15
(B): CH2 γ1 - BEAT CH3 (B) γ1	χ2	n.a.	0.3 ± 0.2	1.6 ± 0.7
Erbitux (IgG1)	KD	0.83 ± 0.01	n.a.	n.a.
	Rmax	140 ± 2	n.a.	n.a.
	χ2	1.57 ± 0.1	n.a.	n.a.
Herceptin (IgG1)	KD	n.a.	4.7 ± 0.4	n.a.
	Rmax	n.a.	112 ± 21	n.a.
	χ2	n.a.	1.15 ± 0.71	n.a.
U1-59 (IgG1)	KD	n.a.	n.a.	1 ± 0.05
	Rmax	n.a.	n.a.	130 ± 6
	χ2	n.a.	n.a.	0.15 ±0.03

TABLE S4

**Dunnett's multiple comparison test**	**Adjusted P Value**	**Significant?**	**Summary**
Herceptin vs. Vehicle	0.13	No	n.s.
anti-HER3 vs. Vehicle	0.93	No	n.s.
Herceptin + anti-HER3 vs. Vehicle	<0.001	Yes	***
Duligotuzumab vs. Vehicle	0.004	Yes	**
BEAT 2/3 vs. Vehicle	<0.001	Yes	***

**Mann Whitney test**	**Adjusted P Value**	**Significant?**	**Summary**
BEAT 2/3 vs. Herceptin	0.0174	Yes	*
BEAT 2/3 vs. anti-HER3	0.0013	Yes	**
BEAT 2/3 vs. Herceptin + anti-HER3	0.0181	Yes	*
